# The Application of Principal Component Analysis on Clinical and Biochemical Parameters Exemplified in Children With Congenital Adrenal Hyperplasia

**DOI:** 10.3389/fendo.2021.652888

**Published:** 2021-08-31

**Authors:** Marie Lindhardt Ljubicic, Andre Madsen, Anders Juul, Kristian Almstrup, Trine Holm Johannsen

**Affiliations:** ^1^Department of Growth and Reproduction, Rigshospitalet, University of Copenhagen, Copenhagen, Denmark; ^2^International Center for Research and Research Training in Endocrine Disruption of Male Reproduction and Child Health (EDMaRC), Rigshospitalet, University of Copenhagen, Copenhagen, Denmark; ^3^Hormone Laboratory, Department of Medical Biochemistry and Pharmacology, Haukeland University Hospital, Bergen, Norway

**Keywords:** principal component analysis, congenital adrenal hyperplasia, CAH, endocrine profiling, treatment efficacy

## Abstract

**Purpose:**

Principal component analysis (PCA) is a mathematical model which simplifies data into new, combined variables. Optimal treatment of pediatric congenital adrenal hyperplasia (CAH) remains a challenge and requires evaluation of all biochemical and clinical markers. The aim of this study was to introduce PCA methodology as a tool to optimize management in a cohort of pediatric and adolescent patients with CAH by including adrenal steroid measurements and clinical parameters.

**Methods:**

This retrospective, longitudinal cohort of 33 children and adolescents with CAH due to 21-hydroxylase deficiency included 406 follow-up observations. PCAs were applied to serum hormone concentrations and compared to treatment efficacy evaluated by clinical parameters.

**Results:**

We provide and describe the first PCA models with hormone parameters denoted in sex- and age-adjusted standard deviation (SD) scores to comprehensibly describe the combined ‘endocrine profiles’ of patients with classical and non-classical CAH, respectively. Endocrine profile scores were predictive markers of treatment efficacy for classical (AUC=92%; accuracy 95%; p=1.8e-06) and non-classical CAH (AUC=80%; accuracy 91%; p=0.004). A combined PCA demonstrated clustering of patients with classical and non-classical CAH by serum 17-hydroxyprogesterone (17-OHP) and dehydroepiandrosterone-sulphate (DHEAS) concentrations.

**Conclusion:**

As an example of the possibilities of PCA, endocrine profiles were successfully able to distinguish between patients with CAH according to treatment efficacy and to elucidate biochemical differences between classical and non-classical CAH.

## Introduction

Congenital adrenal hyperplasia (CAH) is a genetic disorder primarily caused by 21-hydroxylase deficiency. Patients are grouped into classical and non-classical forms according to severity reflecting residual CYP21A2 enzyme activity. The pathophysiology of CAH is largely centered around alterations in the adrenal steroidogenesis, resulting in elevated serum concentrations of 17-hydroxyprogesterone (17-OHP) including the adrenally derived 21-deoxycortisol (1), androgens including 11-oxygenated 19-carbon androgens (2–4) and especially in classical CAH reduced mineralocorticoids and glucocorticoids. Clinically, CAH is associated with increased virilization (5), decreased final height (6), increased body mass index (BMI) (7, 8), and altered blood pressure (BP) (8, 9). As outlined in the most recent consensus statement (5), management of patients with CAH can be challenging, particularly in children and adolescents. For example, under- or overtreatment may stimulate or stunt growth and initiate early pubertal onset. Optimal treatment remains a challenge (1) and relies on evaluation of a growing panel of biochemical and clinical markers (5). Interpreting combinations of these markers introduces complexity, subjectivity, and bias into the patient care.

Principal component analysis (PCA) is a statistical method that can be applied to datasets to obtain a simplified model for stratifying patients or phenotypes by reducing the number of variables (10). In other words, PCA condenses the variables of a dataset into a smaller number of new ‘variables’ named principal components. As such, each principal component is an explanatory variable which represents the relationship between the original variables in the dataset. These principal components provide scores for combinations of variables that explain the most variance within a dataset. Importantly, compared to simpler statistical models and comparisons in which variables are included unweighted, PCA weights the variables according to their relative importance. PCA is therefore a method that can aid in determining key variables and phenotype clusters in large datasets. For example, principal component scores have been applied to enumerate and assign severity scores in newborn screening of CAH (11), in pediatric metabolic syndrome (12), as endocrine profile scores in female puberty (13), and in dietary patterns during childhood (14).

With the general aim of introducing PCA methodology as a method to optimize the interpretation of complex data in diagnostics and management of patients with Differences of Sex Development (DSD), we applied this method to adrenal steroid measurements and clinical parameters in a cohort of pediatric and adolescent patients with CAH.

## Materials and Methods

### Patient Cohort

In this study the total CAH cohort included 33 patients aged 0.3 to 18.9 years. This cohort was described in a recent publication focusing on the use of standard deviation (SD) scores in the management of CAH (15). However, one patient with an additional diagnosis of 45,X/46,XY mosaicism was excluded, as this condition could potentially influence the PCA, while a second patient was excluded due to lack of clinical information.

The current cohort featured 406 visits to the outpatient clinic at the Department of Growth and Reproduction, Rigshospitalet, Copenhagen, from the 33 included patients. The inclusion criteria were genetically verified CAH due to 21-hydroxylase deficiency and measurements of the following four steroid metabolites at each included visit: 17-OHP; dehydroepiandrosterone-sulphate (DHEAS); androstenedione; and testosterone. As previously reported in detail (15), the following information extracted from patient files, when available, was also included: A) clinical information: anthropometry (i.e. height corrected for target height [height SD score minus target height SD score] and body-mass index [BMI]), systolic and diastolic blood pressure, and total glucocorticoid dose per body surface area without regard to timing of dosages throughout the day, and B) biochemical information: serum concentrations of sex-hormone-binding globulin (SHBG), luteinizing hormone (LH), and follicle-stimulating hormone (FSH). Except for glucocorticoid dose, all included variables were expressed as sex- and age-adjusted SD scores to enable comparison of all data entries across sexes and different ages as previously described (15).

Each of the 406 patient visits was deemed a separate observation in the PCA model. The rationale was that this would allow for the full dataset to be exploited by the PCA models and that treatment efficacy is somewhat dynamic, and modulations are made on a visit-to-visit basis in the clinical setting. Thus, for this paper we assumed that the clinical and biochemical variables reflected the current treatment for any given visit. In other words, the PCA model applied the 406 visits unpaired, neglecting that some observations originate from the same patient. Treatment efficacy was evaluated for each visit based on clinical data from routine follow-ups: height minus target height; BMI; and systolic and diastolic blood pressure (all expressed in SD scores). Treatment efficacy was categorized as *optimal* when all variables were between ± 1.5 SD scores, as *sub-optimal* when at least one variable was outside ± 1.5 SD scores, or as *insufficient* when all variables were outside ± 1.5 SD scores. The narrow interval of ± 1.5 SD scores was chosen to ensure that patients within this interval were indeed optimally treated. The applied treatment stratification was objective and observer-independent while allowing us to explore whether the PCA methodology would be able to distinguish between treatment efficacy groups. However, the objectivity also introduced an obvious and crude simplification of an individual’s treatment status. Notably, biochemical markers were only included in the PCA model and were not part of treatment efficacy stratification. Conversely, clinical markers were not included in the PCA models.

### Hormone Assays

Serum 17-OHP and the adrenal androgens (DHEAS, androstenedione, and testosterone) were measured by liquid chromatography tandem-mass spectrometry-based (LC-MS/MS) with inter-assay coefficients of variation (CVs) ranging from 1.4 to 2.5% (15). These androgens were included as they are part of routine follow-up in our clinic. The method has previously been described in detail (16). Serum LH and FSH were analyzed by a time-resolved fluoro-immuno-assay (AutoDELFIA, Perkin Elmer, Turku, Finland) with an inter-assay coefficient of variation (CV) below 5% and a limit of detection (LoD) of 0.05 IU/L for both. Until September 2014, serum SHBG was measured by AutoDELFIA with a CV below 6% and a LoD of 0.23 nM), while it from September 2014 and onwards was measured by a chemiluminescence immunoassay (Access2, Beckman Coulter, Brea, CA, USA) with a LoD of 0.35 nmol/L and a CV below 5%. All AutoDELFIA-derived SHBG results were factored to corresponding Access2 SHBG results after internal method comparison.

### Hormone References Curves

The use of the Generalized Additive Model for Location, Scale, and Shape (GAMLSS) to obtain SD scores has previously been described for height and BMI (17), blood pressure (15), 17-OHP and the adrenal androgens (15), and LH and FSH (18). Based on healthy participants aged 0.2 to 20.0 years of age (19), reference curves were established for SHBG (1068 males, 668 females). The GAMLSS algorithm was applied using *R* (20).

### Principal Component Analysis

Dimension reduction by PCA was applied to sex- and age-adjusted SD scores for the following endocrine variables: 17-OHP, DHEAS, androstenedione, testosterone, SHBG, LH, and FSH. Patient observations with one or more missing hormone measurements were not included. Accordingly, 109 and 93 eligible visits (i.e. observations) from 18 patients with classical CAH and 15 patients with non-classical CAH, respectively, were included. PCA computation was done using the *prcomp()* command in R without variable scaling, as previously described (13). PCA biplots were generated using the *ggbiplot* package in *R* (21).

The PCA output typically consists of five to ten uncorrelated principal components, each explaining a proportion of the total dataset variance. The first principal component typically explains most of the variance, but successive principal components may also detect relevant variance aligning with a topic of interest (e.g. treatment efficacy). In this study, principal components were described in terms of a) proportion of explained variance and b) Eigenvalue (i.e. the principal component standard deviation squared). Only principal components with an Eigenvalue above 1.0 were considered significant (22). Each principal component contains a unique combination of correlation coefficients between the principal component and the input variables. These coefficients were deemed strong if above 0.4.

The correlation coefficients from the different principal components can be used to calculate new, numerical principal components scores (hereafter named endocrine profile scores because only the biochemical markers are included in the PCAs included in this study). In context of the total dataset variance, such endocrine profile scores represent coordinates that tend to cluster related ‘phenotypes’ within a dataset. Thus, endocrine profile scores were applied to distinguish between treatment efficacy groups. The coefficients required to calculate endocrine profile scores equivalent to our current models to detect treatment efficacy are provided in **Supplementary Table S1**.

### Other Statistics

Receiver operating characteristic (ROC) curves were computed using the pROC package in *R* and used to evaluate the ability of individual biochemical markers and the PCA-based endocrine profile scores to distinguish between optimally and insufficiently treated patients. The ROC Youden index was used to determine the optimal biomarker cutoff to distinguish the two groups (23). Also based on ROC, the area under the curve (AUC) was used to evaluate the ability of a variable (i.e. endocrine profile score and individual biochemical markers to distinguish between groups: ≥90%: excellent, ≥80%: good, ≥70: fair, ≥60%: poor, and <60%: fail, as previously detailed (24). Statistical differences between groups were evaluated by Wilcoxon rank sum test (R), corresponding to the Mann Whitney U test (GraphPad Prism).

## Results

In total, 33 children and adolescents with CAH were included (six boys and 27 girls), representing 406 successive visits to the outpatient clinic. Glucocorticoid treatment was administered at all visits by classical CAH patients and in 137 of 187 visits by non-classical CAH patients. Treatment efficacy was evaluated at each visit by SD scores and grouped accordingly into *optimally*, *sub-optimally, or insufficiently* treated, as described in the Methods section. Baseline characteristics of the optimally and insufficiently treated patients are provided in **Table 1**. To minimize misclassification across treatment efficacy groups, we focused on the two treatment extremes and assessed the performance of the listed hormones as biochemical markers (**Table 1**). Notably, optimally treated patients were significantly shorter and leaner than insufficiently treated patients. There was no difference in glucocorticoid doses between the treatment groups (**Table 1**). Optimally treated patients exhibited significantly lower SD scores of serum DHEAS, testosterone, SHBG, and LH, and significantly higher SD scores of serum FSH, while SD scores of serum 17-OHP did not differ between the patient groups. As evident from ROC analyses, no single hormone represented a viable biochemical marker of treatment efficacy.

**Table 1:** Baseline characteristics and biomarker performance for treatment efficacy groups stratified into *optimally* (n=186) and *insufficiently* (n=38) treated patients with congenital adrenal hyperplasia.

Variable	Optimally treated		Insufficiently treated			ROC biomarker performance							
	median (IQR)	n	median (IQR)	n	P-value	AUC (%)	Sens. (%)	Spec. (%)	PPV (%)	NPV (%)	ACC (%)	Ability^a^	Cutoff
***Clinical markers***													
BMI (SDS)	0.15 (-1.17 to 1.28)	156	1.97 (1.03 to 5.11)	35	2.2e-16	–	–	–	–	–	–	–	–
H-TH (SDS)	0.15 (-1.32 to 1.42)	156	2.20 (1.09 to 2.81)	35	2.2e-16	–	–	–	–	–	–	–	–
Diastolic BP (SDS)	0.27 (-1.38 to 1.42)	36	1.64 (-2.64 to 2.82)	5	0.63	–	–	–	–	–	–	–	**-**
Systolic BP (SDS)	0.14 (-1.36 to 1.50)	36	1.51 (-2.99 to 2.70)	5	0.31	–	–	–	–	–	–	–	–
GC per BSA (mg/m^2^)	12.92 (0.00 to 20.31)	109	13.78 (6.44 to 19.55)	29	0.51	54	61	55	84	27	60	poor	13.6
***Biochemical markers***													
17-OHP (SDS)	4.27 (-1.42 to 15.62)	153	4.46 (1.86 to 14.91)	38	0.22	56	34	84	90	24	44	fail	3.15
DHEAS (SDS)	-2.17 (-5.34 to 2.32)	153	-0.59 (-4.57 to 1.18)	38	0.02	63	50	89	95	30	58	fail	-2.17
Androstenedione (SDS)	0.64 (-2.66 to 4.64)	154	1.14 (-1.67 to 3.74)	38	0.06	60	38	89	94	26	48	fail	-0.16
Testosterone (SDS)	0.75 (-2.07 to 9.75)	151	2.42 (-0.74 to 4.79)	38	0.001	67	59	79	92	32	63	poor	1.29
SHBG (SDS)	-0.09 (-2.59 to 2.40)	148	-0.68 (-1.49 to 1.94)	37	0.0006	68	63	81	93	35	67	poor	-0.20
LH (SDS)	0.18 (-1.80 to 3.65)	59	1.50 (0.94 to 3.68)	26	0.008	68	73	59	80	50	69	poor	1.10
FSH (SDS)	-0.28 (-1.91 to 1.69)	58	-1.79 (-3.39 to 0.42)	26	1.4e-05	80	66	85	90	52	71	good	-0.71

SDS, standard deviation score; BMI, body mass index; H-TH, height minus target height; BP, blood pressure; GC per BSA, glucocorticoid dosage per body surface area; 17-OHP, 17-hydroxyprogesterone; DHEAS, dehydroepiandrosterone sulphate; SHBG, sex hormone-binding globulin; ROC, receiver operating characteristics; AUC, area under the curve; sens., sensitivity; spec., specificity; PPV, positive predictive value; NPV, negative predictive value; ACC, accuracy; IQR, interquartile range.^a^The ability of the biochemical markers to distinguish between treatment efficacy groups (optimally vs insufficiently treated); -, ROC analyses were not performed because these markers provided the basis for the stratification of repeated patient observations as optimally or insufficiently treated.

In order to investigate if any combination of these hormones could discriminate between optimally and insufficiently treated patients, we applied PCA to all included hormones. Due to the marked clinical differences between classical and non-classical CAH, a PCA was performed for each CAH group separately. The PCAs produced four principal components for each CAH group, each accounting for substantial variance (Eigenvalues > 1), **Table 2**. The listed correlation coefficients describe the relative contribution of each hormone to a given principal component. In classical CAH, principal component 1 (representing 76% of the total variance) was primarily dominated by 17-OHP with a correlation coefficient of 0.93. In contrast, principal component 1 in non-classical CAH (representing 63% of the total variance) exhibited strong correlation coefficients for 17-OHP (0.60), androstenedione, DHEAS, and testosterone (coefficients between 0.44 and 0.47).

**Table 2:** Endocrine profiles (principal components) based on hormone concentrations expressed as standard deviation scores in classical and non-classical congenital adrenal hyperplasia (CAH) created by principal component analysis.

	Classical CAH (n = 109)				Non-classical CAH (n = 93)			
	PC1	PC2	PC3	PC4	PC1	PC2	PC3	PC4
***Variance***								
Proportion of variance (%)	76.01	14.56	3.16	2.45	62.60	14.74	10.49	5.10
Cumulative variance (%)	76.01	90.57	93.73	96.20	62.60	77.34	87.83	92.90
Eigenvalue (SD^2^)	37.50	7.18	1.56	1.22	13.73	3.23	2.30	1.11
***Correlation coefficients***								
17-OHP	0.93	-0.29	0.06	-0.05	-0.60	-0.78	-0.02	-0.10
Androstenedione	0.26	0.44	-0.05	0.04	-0.45	0.19	0.18	0.21
Testosterone	0.22	0.50	-0.17	0.38	-0.44	0.29	-0.01	0.54
DHEAS	0.10	0.51	0.13	-0.13	-0.47	0.51	-0.21	-0.60
SHBG	-0.05	-0.04	0.18	0.88	0.03	0.04	0.02	0.45
LH	-0.01	0.45	0.01	-0.25	-0.12	0.12	0.59	0.10
FSH	0.01	-0.06	-0.96	0.07	0.04	-0.01	0.76	-0.29

PC, principal component; SD, standard deviation; 17-OHP, 17-hydroxyprogesterone; DHEAS, dehydroepiandrosterone-sulphate; SHBG, sex hormone-binding globulin; LH, luteinizing hormone; FSH, follicle-stimulating hormone.

**Figure 1:**
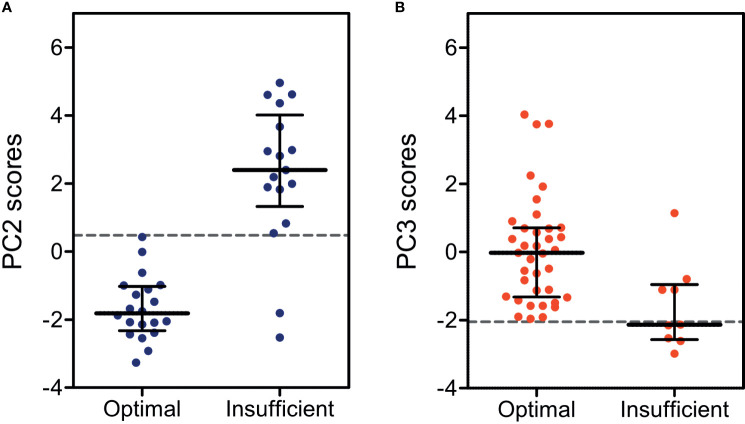
Dot plots for endocrine profile scores calculated from **(A)** principal component 2 (PC2) in children and adolescents with classical CAH (red) and from **(B)** principal component 3 (PC3) in non-classical CAH (blue), both stratified by *optimal* and *insufficient* treatment efficacy. Dashed lines indicate cutoffs for the endocrine profile scores as determined by receiver operating curves.

Beyond describing the endocrine variance in the dataset, we also investigated whether the obtained principal components (**Table 2**) would be able to establish patient clustering with regard to treatment efficacy. We therefore applied endocrine profile scores for each patient observation to examine if these were better markers of treatment efficacy groups than the individual biochemical markers. The PCA produced four informative principal components (Eigenvalue > 1, listed in **Table 2**) and four corresponding endocrine profile scores were obtained for each patient. By applying the scores directly in standard ROC analyses, we found that principal component 2 was the optimal marker of treatment efficacy in classical CAH and principal component 3 was the optimal marker in non-classical CAH (**Table 3**). With cutoffs of 0.48 in classical CAH and -2.05 in non-classical CAH, respectively (**Table 3**), the discrimination between optimally and insufficiently treated patients, with virtually no overlap in classical CAH, was visible in **Figure 1**. The accuracies of the endocrine profiles scores (classical CAH: 95%, non-classical CAH: 91%) reflected that these were better markers of treatment efficacy than the best individual biochemical marker (FSH: 71%). An example of the calculation of endocrine profile score and subsequent evaluation of treatment efficacy is provided in **Supplementary Table S1**.

**Table 3:** Performance of endocrine profile scores (i.e. PC scores) obtained by principal component analyses to distinguish between optimally and insufficiently treated patients with congenital adrenal hyperplasia (CAH).

ROC specifications	Classification of treatment efficacy	
	Classical CAH (n = 37^a^)	Non-classical CAH (n = 46^b^)
	PC score (PC2)	PC score (PC3)
AUC (%)	93	80
Sensitivity (%)	100	100
Specificity (%)	88	56
PPV (%)	91	90
NPV (%)	100	100
Accuracy (%)	95	91
Ability^c^	Excellent	Good
Cutoff score	0.48	-2.05
P-value	1.8e-06	0.004

ROC, receiver operating characteristics; PC, principal component; AUC, area under the curve; PPV, positive predictive value; NPV, negative predictive value; ^a^37 visits = 20 optimally treated + 17 insufficiently treated; ^b^46 visits = 37 optimally treated + 9 insufficiently treated; ^c^The ability of the PC scores to distinguish between treatment efficacy groups (optimally vs insufficiently treated). Biomarker performance was evaluated by receiver operating characteristics analyses.

**Figure 2:**
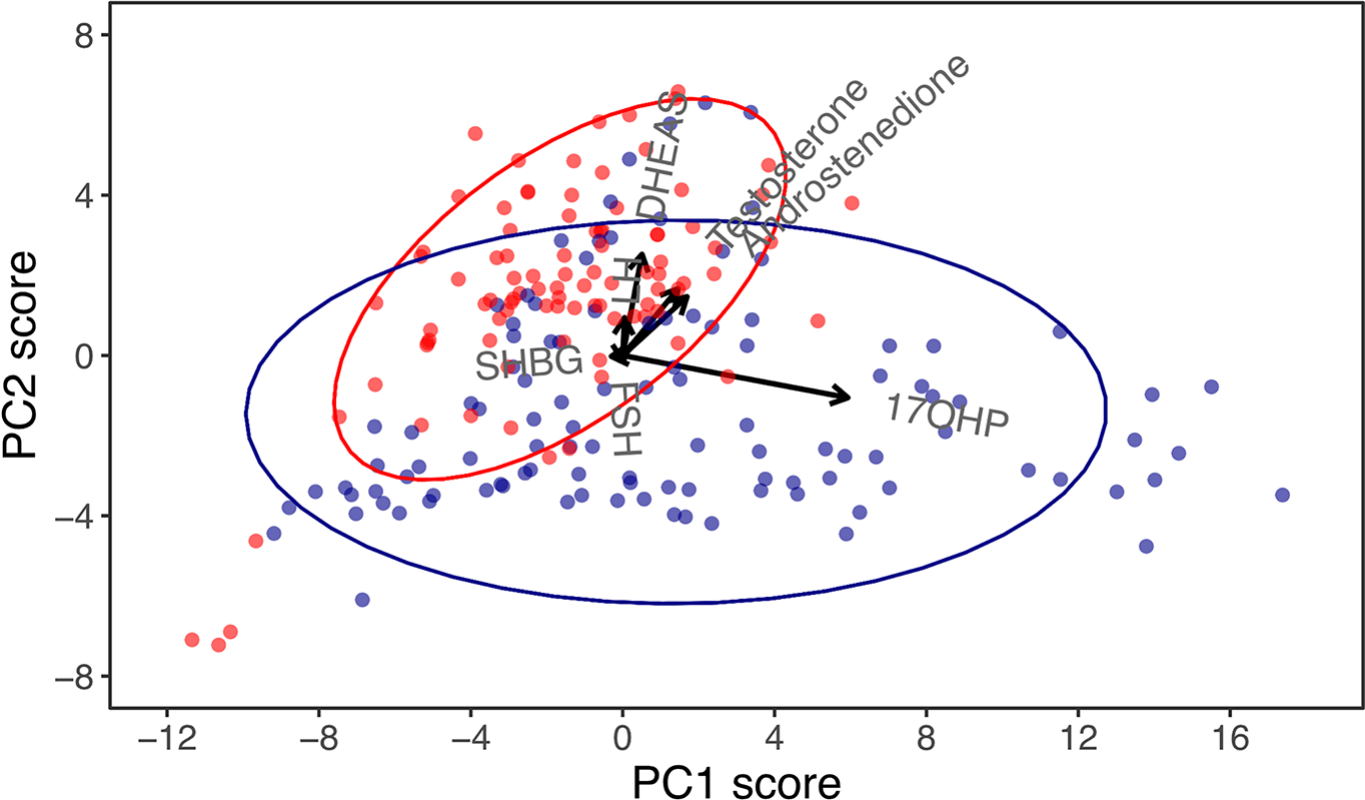
Principal component (PC) biplot for PC1 and PC2 in children and adolescents with congenital adrenal hyperplasia (CAH), accounting for 87% of the dataset variance. Arrows are vectors that represent the correlation coefficients of biochemical markers with principal components, and these should be interpreted horizontally for PC1 and vertically for PC2. While 17-hydroxyprogesterone (17-OHP) was almost aligned in the horizontal plane, indicating a strong correlation with PC1, dehydroepiandrosterone-sulphate (DHEAS) was almost aligned vertically, indicating a strong correlation with PC2. Dots represent visits of patients with classical (blue) and non-classical (red) CAH.

Combining the classical and non-classical CAH datasets in a new PCA allowed for a different overview of the data not aimed at discriminating treatment efficacy patients. With the aim of investigating the underlying biochemistry of CAH, a so-called biplot featuring the two principal components accounting for the most dataset variance was created (**Figure 2**). The biplot revealed a perpendicular relationship between the 17-OHP and DHEAS vectors. This was also evident in the respective correlation coefficients for 17-OHP in principal component 1 (0.93) and DHEAS in principal component 2 (0.69). In the biplot, patients with classical and non-classical CAH, respectively, clustered. This sparked a rationale for using the 17-OHP/DHEAS ratio to separate classical and non-classical CAH. Using ROC curves, the 17-OHP/DHEAS ratio was deemed a ‘good’ discriminator between the two CAH groups with AUC, sensitivity, specificity, PPV, NPV, and accuracy ranging from 81-89%, p=2.2e-16 (data not shown).

## Discussion

In this pilot study, we introduce the simultaneous and combined evaluation of several hormones by use of PCAs in a cohort of children and adolescents with CAH and demonstrate the immediate benefits of this method of simplification. Namely, we show that an endocrine profile score produced by PCA was able to distinguish between optimally and insufficiently treated patients with classical CAH, although conclusions are limited by the small dataset. Thus, further studies applying PCA in CAH cohorts are warranted.

Within the field of Differences of Sex Development, new clinical and biochemical markers continue to emerge, making it increasingly difficult for clinicians to reconcile the many markers at the same time. The superiority of PCA compared to simpler, traditional statistical methods is best described by its ability to reduce complex data by identifying redundant variables and uncover patient clusters that may correspond to clinically relevant phenotypes. PCA has previously been successfully used in endocrinology, for example to enhance detection of CAH in newborns (11) and pubertal onset in girls (13) and to distinguish endocrine profile clusters with implications for pubertal timing in girls (25). Moreover, PCA enabled the conclusion that the metabolic profile of polycystic ovarian syndrome is neither intrinsic nor specific (26).

Applying PCA to our dataset revealed possible new approaches to the management of patients with CAH, although conclusions are limited due to the small dataset. Nonetheless, PCA allowed for principal components to be created that included and weighed all hormone SD scores that were included in the model. The corresponding endocrine profile scores (calculated from principal component 2 in classical CAH and from principal component 3 in non-classical CAH) were able to distinguish between optimally and insufficiently treated patients. The fact that these scores outperformed the individual biochemical markers highlights the strength of combining hormones into a single variable by PCA. Theoretically, the endocrine profile scores could be applied in any outpatient clinic, enabling the physician to determine whether e.g. adjustment of glucocorticoid treatment in a CAH patient is needed at any given time. By simple calculation of endocrine profile scores, subsequent evaluation of treatment efficacy by relevant cutoffs is theoretically possible in other centers as well. Ideally, each center should calculate its own cutoffs based on local data, but the cutoffs presented in this study are based on sex and age-adjusted SD scores, which allows for comparison across sexes, ages, and centers. Thus, in the real world, the current cutoffs may aid clinicians from other centers as well if applied with caution. Despite this, we also recognize the need for a larger and more homogenous dataset to fully optimize management of CAH patients.

In line with this, our dataset was small, and the outcome of treatment efficacy was limited partly by the simplistic and somewhat artificial groups and partly by the availability of clinical information for each visit, making misclassification a possibility. Furthermore, we were unable to include clinical information such as virilization, bone ages, and other biochemical markers (renin, 21-deoxycortisol, and 11-oxygenated 19-carbon steroids), which would arguably have improved stratification and optimized our PCA results.

Examining the two PCA outputs from the patients with classical and non-classical CAH, respectively, revealed integral differences in terms of endocrine constellations. In classical CAH, 17-OHP was strongly correlated to the primary principal component, whereas in non-classical CAH both 17-OHP and DHEAS were strongly correlated to the primary principal component. Thus, 17-OHP was shown to dominate both primary principal components, but it did not discriminate between optimally and insufficiently treated patients. In line with current guidelines (5), this highlights that 17-OHP is not a viable single marker of treatment efficacy. Similarly, DHEAS played a larger role in the variation between patients with non-classical CAH than between patients with classical CAH. These differences in 17-OHP and DHEAS were also visible when combining all patients (classical as well as non-classical) in a PCA-derived biplot that clustered the two CAH groups. The differences in 17-OHP and DHEAS were further highlighted by the ability of their ratio to distinguish between patients with classical and non-classical CAH. However, due to the majority of the patients undergoing treatment in our cohort, this ratio cannot be directly applied in CAH diagnostics. Though the ratio may not be clinically relevant, the process of deriving the ratio and the finding altogether demonstrates that PCA methodology can be used to illuminate the underlying biochemistry. This use of PCA has also previously been reported in patients with polycystic ovaries (26). By example, we therefore demonstrate how PCA can introduce new markers and ratios of possible clinical interest in future research projects on patients with CAH.

The methodological strengths of this pilot study included 1) the introduction of a powerful statistical method in the context of CAH; 2) the use of SD scores for all clinical and biochemical markers which allowed for PCA models and comparisons across sex and age and without consequent loss of sample size; and 3) the use of liquid chromatography tandem-mass spectrometry in the analysis of 17-OHP and adrenal androgens. The limitations included 1) the dataset was small and each observation in the PCA model equated a follow-up visit introducing possible biases, i.e. insufficiently treated or patients at higher risk of complications may have been overrepresented due to more visits as a direct consequence; 2) in 73% of visits by patients with non-classical CAH, patients received glucocorticoid treatment which may be an overrepresentation of those needing treatment; 3) stratification of CAH patients according to treatment efficacy based on equally weighted clinical parameters (anthropometry and blood pressure) was simplistic and may not accurately reflect true treatment efficacy; 4) misclassification between treatment efficacy groups was also a possibility, and the largest group (*suboptimal*) was not analyzed in ROC analyses; 5) treatment efficacy classification did not include virilization status, pubertal status or bone age due to lack of uniformly collected data; 6) measurements of C-19 oxygenated steroids and 21-deoxycortisol were not included as they were not measured; 7) measurements of renin were not included due to lack of reference ranges and thereby lack of SD scores; and 8) blood sampling was done during the opening hours of the outpatient clinic without regard to medicine intake.

In conclusion, with the aim of introducing a combined and simultaneous evaluation of several hormones, we described and applied PCA in a cohort of children and adolescents with CAH. Via multiple hormones reduced to endocrine profile scores, we were able to classify optimally and insufficiently treated patients with CAH. By use of PCA-derived endocrine profile scores and cutoffs, this approach is directly adaptable into a clinical setting. Moreover, the PCA model enabled us to elucidate the relative importance of individual hormones in patients with CAH. We therefore propose that PCA be applied as a tool in future research on larger DSD cohorts.

## Data Availability Statement

The datasets presented in this article are not readily available because of data protection laws. However, the codes used in R are available upon request. Requests to access the datasets should be directed to trine.holm.johannsen@regionh.dk.

## Ethics Statement

This study involving human participants were reviewed and approved by the Danish Patient Safety Authority (no. 3-3013-1376/1/) and the Danish Data Protection Agency (no. 2015-235, I-Suite no. 04204). Written informed consent from the participants’ legal guardian/next of kin was not required to participate in this study in accordance with the national legislation and the institutional requirements.

## Author Contributions

ML and TJ took part in the study design, execution, analysis, manuscript drafting and critical discussion. AM, AJ, and KA took part in the execution, analysis, manuscript drafting and critical discussion. All authors contributed to the article and approved the submitted version.

## Funding

ML was funded by the Absalon Foundation.

## Conflict of Interest

The authors declare that the research was conducted in the absence of any commercial or financial relationships that could be construed as a potential conflict of interest.

## Publisher’s Note

All claims expressed in this article are solely those of the authors and do not necessarily represent those of their affiliated organizations, or those of the publisher, the editors and the reviewers. Any product that may be evaluated in this article, or claim that may be made by its manufacturer, is not guaranteed or endorsed by the publisher.

## References

[1] Merke DP Auchus RJ. Congenital Adrenal Hyperplasia Due to 21-Hydroxylase Deficiency. N Engl J Med (2020) 383(13):1248–61. 10.1056/NEJMra1909786 32966723

[2] Turcu AF Nanba AT Chomic R Upadhyay SK Giordano TJ Shields JJ. Adrenal-Derived 11-Oxygenated 19-Carbon Steroids Are the Dominant Androgens in Classic 21-Hydroxylase Deficiency. Eur J Endocrinol (2016) 174(5):601–9. 10.1530/EJE-15-1181 PMC4874183 26865584

[3] Kamrath C Wettstaedt L Boettcher C Hartmann MF Wudy SA. Androgen Excess Is Due to Elevated 11-Oxygenated Androgens in Treated Children With Congenital Adrenal Hyperplasia. J Steroid Biochem Mol Biol (2018) 178:221–8. 10.1016/j.jsbmb.2017.12.016 29277706

[4] Jha S Turcu AF Sinaii N Brookner B Auchus RJ Merke DP. 11-Oxygenated Androgens Useful in the Setting of Discrepant Conventional Biomarkers in 21-Hydroxylase Deficiency. J Endocr Soc (2020) 5(2):1–9. 10.1210/jendso/bvaa192 PMC7796775 33447690

[5] Speiser PW Arlt W Auchus RJ Baskin LS Conway GS Merke DP. Congenital Adrenal Hyperplasia Due to Steroid 21-Hydroxylase Deficiency: An Endocrine Society* Clinical Practice Guideline. J Clin Endocrinol Metab (2018) 103(11):4043–88. 10.1210/jc.2018-01865 PMC6456929 30272171

[6] Muthusamy K Elamin MB Smushkin G Murad MH Lampropulos JF Elamin KB. Adult Height in Patients With Congenital Adrenal Hyperplasia: A Systematic Review and Metaanalysis. J Clin Endocrinol Metab (2010) 95(9):4161–72. 10.1210/jc.2009-2616 20823467

[7] Völkl TMK Simm D Beier C Dörr HG. Obesity Among Children and Adolescents With Classic Congenital Adrenal Hyperplasia Due to 21-Hydroxylase Deficiency. Pediatrics (2006) 117(1):e98–105. 10.1542/peds.2005-1005 16396852

[8] Mooij CF van Herwaarden AE Sweep FCGJ Roeleveld N de Korte CL Kapusta L. Cardiovascular and Metabolic Risk in Pediatric Patients With Congenital Adrenal Hyperplasia Due to 21 Hydroxylase Deficiency. J Pediatr Endocrinol Metab (2017) 30(9):957–66. 10.1515/jpem-2017-0068 28787274

[9] Roche EF Charmandari E Dattani MT Hindmarsh PC. Blood Pressure in Children and Adolescents With Congenital Adrenal Hyperplasia (21-Hydroxylase Deficiency): A Preliminary Report. Clin Endocrinol (Oxf) (2003) 58(5):589–96. 10.1046/j.1365-2265.2003.01757.x 12699440

[10] Ringnér M. What Is Principal Component Analysis? Nat Biotechnol (2008) 26(3):303–4. 10.1038/nbt0308-303 18327243

[11] Lasarev MR Bialk ER Allen DB Held PK. Application of Principal Component Analysis to Newborn Screening for Congenital Adrenal Hyperplasia. J Clin Endocrinol Metab (2020) 105(8):e2930–40. 10.1210/clinem/dgaa371 32525982

[12] Katzmarzyk PT Pérusse L Malina RM Bergeron J Després J-P Bouchard C. Stability of Indicators of the Metabolic Syndrome From Childhood and Adolescence to Young Childhood: The Québec Family Study. J Clin Epidemiol (2001) 54(2):190–5. 10.1016/S0895-4356(00)00315-2 11166535

[13] Madsen A Bruserud IS Bertelsen B-E Roelants M Oehme NHB Viste K. Hormone References for Ultrasound Breast Staging and Endocrine Profiling to Detect Female Onset of Puberty. J Clin Endocrinol Metab (2020) 105(12):e4886–95. 10.1210/clinem/dgaa679 PMC7571452 32961560

[14] Günther ALB Schulze MB Kroke A Diethelm K Joslowski G Krupp D. Early Diet and Later Cancer Risk: Prospective Associations of Dietary Patterns During Critical Periods of Childhood With the GH-IGF Axis, Insulin Resistance and Body Fatness in Younger Adulthood. Nutr Cancer (2015) 67(6):877–92. 10.1080/01635581.2015.1056313 26226486

[15] Clausen CS Ljubicic ML Main KM Andersson A-M Petersen JH Frederiksen H. Congenital Adrenal Hyperplasia in Children: A Pilot Study of Steroid Hormones Expressed as Sex- and Age-Related Standard Deviation Scores. Horm Res Paediatr (2020) 93(4):226–38. 10.1159/000509079 33017824

[16] Søeborg T Frederiksen H Johannsen TH Andersson A-M Juul A. Isotope-Dilution TurboFlow-LC-MS/MS Method for Simultaneous Quantification of Ten Steroid Metabolites in Serum. Clin Chim Acta (2017) 468:180–6. 10.1016/j.cca.2017.03.002 28263736

[17] Tinggaard J Aksglaede L Sørensen K Mouritsen A Wohlfahrt-Veje C Hagen CP. The 2014 Danish References From Birth to 20 Years for Height, Weight and Body Mass Index. Acta Paediatr (2014) 103(2):214–24. 10.1111/apa.12468 24127859

[18] Ljubicic ML Jespersen K Aksglaede L Hagen CP Petersen JH Andersen HR. The LH/FSH Ratio Is Not a Sex-Dimorphic Marker After Infancy: Data From 6417 Healthy Individuals and 125 Patients With Differences of Sex Development. Hum Reprod (2020) 35(10):2323–35. 10.1093/humrep/deaa182 32976602

[19] Søeborg T Frederiksen H Mouritsen A Johannsen TH Main KM Jørgensen N. Sex, Age, Pubertal Development and Use of Oral Contraceptives in Relation to Serum Concentrations of DHEA, DHEAS, 17α-Hydroxyprogesterone, Δ4-Androstenedione, Testosterone and Their Ratios in Children, Adolescents and Young Adults. Clin Chim Acta (2014) 437:6–13. 10.1016/j.cca.2014.06.018 24976611

[20] Rigby RA Stasinopoulos DM. Generalized Additive Models for Location, Scale and Shape. J R Stat Soc Ser C (2005) 54(3):507–54. 10.1111/j.1467-9876.2005.00510.x

[21] Wickham H. Ggplot2: Elegant Graphics for Data Analysis. 2nd ed. Cham: Springer International Publishing (2016).

[22] Jackson DA. Stopping Rules in Principal Components Analysis: A Comparison of Heuristical and Statistical Approaches. Ecology (1993) 74(8):2204–14. 10.2307/1939574. 10.2307/1939574

[23] Hajian-Tilaki K. Receiver Operating Characteristic (ROC) Curve Analysis for Medical Diagnostic Test Evaluation. Casp J Intern Med (2013) 4(2):627–35. PMC3755824 24009950

[24] Safari S Baratloo A Elfil M Negida A. Evidence Based Emergency Medicine; Part 5 Receiver Operating Curve and Area Under the Curve. Emerg (Tehran Iran) (2016) 4(2):111–3. PMC4893763 27274525

[25] Fassler CS Gutmark-Little I Xie C Giannini CM Chandler DW Biro FM. Sex Hormone Phenotypes in Young Girls and the Age at Pubertal Milestones. J Clin Endocrinol Metab (2019) 104(12):6079–89. 10.1210/jc.2019-00889 PMC6821200 31408174

[26] Dewailly D Pigny P Soudan B Catteau-Jonard S Decanter C Poncelet E. Reconciling the Definitions of Polycystic Ovary Syndrome: The Ovarian Follicle Number and Serum Anti-Müllerian Hormone Concentrations Aggregate With the Markers of Hyperandrogenism. J Clin Endocrinol Metab (2010) 95(9):4399–405. 10.1210/jc.2010-0334 20610596

