# Supplementary material for: The Application of Principal Component Analysis on Clinical and Biochemical Parameters Exemplified in Children With Congenital Adrenal Hyperplasia
Source: Front Endocrinol (Lausanne). 2021 Aug 31;12:652888. doi: 10.3389/fendo.2021.652888 (PMC8438425; doi:10.3389/fendo.2021.652888)
Supplement: Supplementary file 1 [file Table_1.docx]

**Supplementary Table 1.** Principal component specifications that enable scoring of individual patients to detect treatment efficacy based on principal component analyses of biochemical markers in classical and non-classical congenital adrenal hyperplasia (CAH).

|  | **Classical CAH (PC2)** | |  | **Non-classical CAH (PC3)** | |
| --- | --- | --- | --- | --- | --- |
| **PCA variable** | **Center coefficient** | **Correlation coefficient** |  | **Center coefficient** | **Correlation coefficient** |
| 17-OHP | 7.53 | -0.29 |  | 3.91 | -0.02 |
| Androstenedione | 0.95 | 0.44 |  | 1.06 | 0.18 |
| Testosterone | 1.40 | 0.50 |  | 1.79 | -0.01 |
| DHEAS | -2.95 | 0.51 |  | -0.40 | -0.21 |
| SHBG | -0.44 | -0.04 |  | -0.23 | 0.02 |
| FSH | -0.41 | -0.06 |  | -0.37 | 0.76 |
| LH | 0.62 | 0.45 |  | 0.73 | 0.59 |
| endocrine profile score cutoff | *insufficient* > 0.48 > *optimal* | |  | *insufficient* < -2.05 < *optimal* | |

Abbreviations: PC: principal component; PCA: PC analysis; SHBG: sex hormone-binding globulin; DHEAS: dehydroepiandrosterone-sulphate; 17OHP: 17-hydroxyprogesterone; FSH: follicle-stimulating hormone; LH: luteinizing hormone.

This table provides the specifications required to calculate endocrine profile scores (i.e. PC scores) for new patient observations. The calculation is outlined below. Briefly, the relevant center coefficient is subtracted from the patient hormone concentration standard deviation (SD) score, and this number is multiplied by the associated correlation coefficient for each hormone. The sum is the endocrine profile score.

For example, to calculate the endocrine profile score based on center and correlation coefficients from PC2 a classical CAH patient exhibiting a standard deviation score of 2.0 for all hormones listed above, apply the following equation:

*Σ ([hormone SD score – center coefficient] * correlation coefficient):*

Testosterone component: ([2.0 - 1.4] * 0.50)

SHBG component: + ([2.0 - (-0.44]) * -0.04)

DHEAS component: + ([2.0 - (-2.95]) * 0.51)

Androstenedione component: + ([2.0 - 0.95] * 0.44)

17-OHP component: + ([2.0 - 7.53] * -0.29)

FSH component: + ([2.0 - (-0.41]) * -0.06)

LH component: + ([2.0 - 0.62] * 0.45) .

**endocrine profile score from PC2 = 5.27**

Interpretation: A value of 5.27 is greater than the cutoff of 0.48 indicating *insufficient* treatment efficacy.
